# Comparative Analysis of Vitamin D, Folic Acid, and Antioxidant Minerals in Various Stages of Lung Cancer

**DOI:** 10.7759/cureus.71696

**Published:** 2024-10-17

**Authors:** Rajyalakshmi Gogineni, Suresh Arumugam, Natrajan Muninathan, Kuppusamy Baskaran

**Affiliations:** 1 Department of Biochemistry, Meenakshi Medical College Hospital and Research Institute, Kanchipuram, IND; 2 Department of Microbiology, Meenakshi Medical College Hospital and Research Institute, Kanchipuram, IND

**Keywords:** antioxidants, cancer stages, copper, folic acid, lung cancer, magnesium, nutrient analysis, vitamin d, zinc

## Abstract

Background: Lung cancer continues to be one of the most common causes of death due to lung malignancies globally. Emerging research suggests that vitamins and trace minerals, particularly antioxidants, may play a role in cancer progression and treatment outcomes. This study conducts a comparative analysis of vitamin D, folic acid, and trace minerals (copper, zinc, and magnesium) in various stages of lung cancer patients.

Methods: A cross-sectional study was conducted with 160 lung cancer patients, categorized into four stages (Stage 1 to Stage 4). Standardized biochemical assays, such as chemiluminescent immunoassay (CLIA), enzyme-linked immunosorbent assay (ELISA), and atomic absorption spectroscopy (AAS), were used to measure the levels of vitamin D, folic acid, copper (Cu), zinc (Zn), and magnesium (Mg) in the blood. The nutrient levels were compared across stages to investigate any significant variations.

Results: Vitamin D levels decreased significantly as lung cancer progressed, with Stage 1 showing the highest mean level (33 ng/mL) and Stage 4 the lowest (8 ng/mL). Folic acid levels fluctuated, showing a general decrease in the advanced stages, with some variations in the intermediate stages. Copper levels showed individual variability without a consistent trend across stages. Zinc levels were higher in early-stage patients and decreased as cancer progressed. Magnesium levels remained relatively stable across all stages.

Conclusion: This comparative analysis highlights the potential significance of monitoring vitamin D, folic acid, and trace minerals in lung cancer patients across different stages. The results suggest that these nutrients may play a role in the progression of lung cancer and could serve as biomarkers for disease staging.

## Introduction

Overall, lung cancer remains one of the most prevalent cancers with high mortality globally [1], representing a significant proportion of total cases. As the disease progresses, a person’s nutritional status, environmental exposure, and genetic predisposition all have an impact on lung cancer [2]. Recent research has increasingly focused on the role of micronutrients, especially vitamins and trace minerals, in cancer prevention and treatment [3].

Vitamin D, a fat-soluble vitamin crucial for calcium homeostasis and bone health, has attracted attention for its potential role in modulating cancer progression [4]. Adequate levels of vitamin D have been linked to tumor suppression, promotion of cellular differentiation, and induction of apoptosis in cancer cells [5].

Folic acid, an essential B vitamin, plays a critical role in DNA synthesis, repair, and methylation, which are fundamental processes for maintaining normal cellular function. In the context of cancer biology, its role becomes more complex [6]. Cancer cells, characterized by rapid and uncontrolled division, have an increased demand for DNA synthesis, making folic acid crucial for both healthy and cancerous cells. However, the impact of folic acid on cancer development is dual-faceted. A deficiency in folic acid can lead to impaired DNA synthesis and repair, resulting in chromosomal instability and mutations that may trigger cancerous transformations. On the other hand, an excess of folic acid, particularly in individuals with pre-cancerous or cancerous cells, can accelerate tumor growth by providing the necessary resources for the rapid proliferation of these cells [6, 7]. Studies suggest that while folic acid supplementation can be protective in folate-deficient individuals, it may promote tumor progression in those with established cancers. This complex relationship underscores the need for a balanced approach to folic acid intake, where both deficiency and excess are avoided, and highlights the importance of personalized nutrition strategies to manage cancer risk and progression [6-9].

Trace minerals such as copper (Cu), zinc (Zn), and magnesium (Mg) are essential for maintaining cellular homeostasis and protecting against oxidative stress [10-11]. These antioxidant minerals serve as cofactors for various enzymatic processes and are vital for immune function and DNA repair [12]. Dysregulation of these minerals has been implicated in a variety of diseases, including cancer [13].

Given the significant roles of these micronutrients, this study aims to compare the serum levels of vitamin D, folic acid, copper, zinc, and magnesium in lung cancer patients across different stages of the disease. By analyzing how these nutrient levels vary across stages, this research seeks to provide insights into their potential roles in lung cancer progression and explore how nutritional factors may interact with the disease process. Understanding these relationships could open avenues for new therapeutic strategies aimed at improving outcomes for lung cancer patients.

## Materials and methods

Study design

The present cross-sectional study was performed to assess and compare serum levels of vitamin D, folic acid, and trace minerals (copper, zinc, and magnesium) in various stages of lung cancer patients.

Place of Study

The study was carried out at the Tammannagari Ramakrishna Reddy (TRR) Institute of Medical Sciences, Hyderabad, and the RVM Institute of Medical Sciences and Research Center, Mulugu, Telangana. Our study is approved by the Institutional Ethics Committee of TRR Institute of Medical Sciences (TRRIMS/Research/015/2022) Written informed consent was acquired from every participant prior to their inclusion into the study. The anonymity and confidentiality of the participants were preserved.

Study Period

The study was conducted over a period of 30 months, from May 2022 to May 2024.

Study Population

A total of 280 patients were initially screened for participation in this study at the RVM Institute of Medical Sciences and the TRR Institute of Medical Sciences, between May 2022 and May 2024. The screening process involved evaluating patients with suspected or confirmed lung cancer who were receiving treatment or being monitored in the respective oncology departments.

Screening process

The initial screening involved reviewing medical records and conducting clinical assessments to ensure the eligibility of potential participants. During this phase, patients were assessed based on clinical, pathological, and radiological criteria to confirm the diagnosis of lung cancer. Those with other types of cancers or significant comorbidities that could interfere with the study outcomes were excluded. Following this initial screening, 160 eligible lung cancer patients were included in the final study.

Sample size calculation

The sample size was calculated using a power analysis based on previous studies examining serum nutrient levels in cancer patients. The calculations aimed to detect a statistically significant difference in nutrient levels (such as vitamin D, folic acid, and trace minerals) across different stages of lung cancer with 80% power and a 5% significance level (alpha = 0.05). An expected effect size (Cohen’s d) of 0.5 was used, considering moderate variability in nutrient levels across cancer stages. Based on these parameters, a minimum of 140 patients was required. To account for potential dropouts or exclusions during the study, 160 patients were ultimately included, ensuring robust statistical power for subgroup analyses.

Inclusion criteria

Confirmed diagnosis of lung cancer based on histopathological findings, clinical presentation, and imaging results (e.g., CT, PET scans); patients at various stages of lung cancer (Stage 1 to Stage 4) as determined by clinical and pathological staging, based on the American Joint Committee on Cancer (AJCC) criteria; patients receiving treatment at the RVM Institute of Medical Sciences or TRR Institute of Medical Sciences; and patients who provided informed consent to participate in the study and were willing to undergo the required blood tests and assessments.

Exclusion criteria

Patients with cancers other than lung cancer or with mixed malignancies were excluded to ensure the study’s focus on lung cancer-specific nutrient variations; patients with severe comorbid conditions (such as uncontrolled diabetes, liver disease, or chronic kidney disease) that could affect serum nutrient levels independently of lung cancer were excluded; patients who did not provide informed consent or were unable to comply with study procedures were not included in the final analysis.

Final sample

After the initial screening, 120 patients were excluded from the study. These exclusions were primarily due to: 40 patients had other cancers or were found to have metastases from non-lung primary tumors; 50 patients had severe comorbidities (e.g., end-stage renal disease, severe liver dysfunction); and 30 patients did not meet the inclusion criteria due to incomplete medical records, refusal to provide consent, or inability to participate in follow-up assessments.

Thus, 160 patients with confirmed lung cancer at various stages were included in the study. These patients were categorized into four groups corresponding to the clinical stages of lung cancer (Stage 1 to Stage 4). The final study population was balanced in terms of age, sex, and other demographic variables, with efforts made to ensure that the participants represented a wide range of lung cancer severity.

Cancer Staging (Stage 1 to 4)

Lung cancer staging for the study participants was performed based on the AJCC TNM staging system, which is widely recognized for lung cancer classification. This system categorizes lung cancer based on three factors:

T (tumor size and extent): Assessed via imaging techniques such as CT or MRI to determine the size and local spread of the tumor.

N (node involvement): Evaluation of lymph node involvement using PET-CT scans or biopsy to identify if cancer has spread to regional lymph nodes.

M (metastasis): Assessment of distant metastasis through imaging (e.g., bone scans, PET scans) and laboratory tests to determine if the cancer has spread to distant organs.

Based on this TNM classification: Stage 1 represents early-stage, localized lung cancer with no lymph node involvement or metastasis; Stages 2 and 3 represent the more advanced local and regional spread of the tumor, including the involvement of nearby lymph nodes; Stage 4 represents advanced disease with distant metastasis. This staging process ensured accurate categorization of patients, allowing for the comparison of nutrient levels at different stages of lung cancer progression.

Sample collection

Serum samples were collected using sodium heparin or lithium heparin as anticoagulants at both the TRR Institute of Medical Sciences, Hyderabad, and the RVM Institute of Medical Sciences and Research Center, Mulugu, Telangana. Blood samples were carefully taken, following standard procedures to ensure safety and keep the samples intact. The samples were then centrifuged at 4,000 rpm to separate the serum, which was stored at -80°C for later analysis.

Biochemical analysis

Vitamin D

Serum levels were measured using a chemiluminescent immunoassay (CLIA) method. Briefly, this assay follows a competitive binding immune enzymatic method where 25(OH)D is released from the vitamin D-binding protein and forms an immunocomplex. The resulting chemiluminescent reaction is measured, and the relative light units generated are inversely proportional to the 25(OH)D concentration. The assay was standardized against national standards, ensuring traceability and accuracy. Quality control samples showed a mean bias of less than 5%. After processing, serum samples were stored at -80°C until further analysis. This procedure ensures accurate and reliable estimation of vitamin D levels, critical for assessing the vitamin D status of the study participants [14].

Folic Acid

Serum folic acid levels were determined using an enzyme-linked immunosorbent assay (ELISA). The assay is conducted on disposable microfluidic chips, which contain eight parallel microchannels etched into a polyimide substrate. These channels are pre-coated with folic acid-BSA conjugate (FA-BSA), and the surface is blocked to prevent non-specific binding. In the competitive immunoassay step, a mixture of the folic acid sample and an alkaline phosphatase-labeled anti-folic acid antibody (AcFA-ALP) is introduced into the microchannels. The folic acid in the sample competes with the FA-BSA coating for available binding sites on the antibody. After incubation, the enzymatic substrate (p-aminophenyl phosphate) is introduced, and the enzyme-labeled antibody catalyzes a reaction, producing a measurable signal. This signal, typically detected amperometrically, is proportional to the folic acid concentration in the sample. Calibration curves generated from known folic acid concentrations are used to quantify the folic acid levels in the sample, providing a sensitive and efficient method for folic acid estimation [15].

Copper and Zinc

For the estimation of copper, both flame atomic absorption spectrophotometry (FAAS) and a colorimetric method were employed. Serum samples were prepared by diluting 300 µL of the sample in 600 µL of deionized water before analysis. In the FAAS method, the copper content was measured at a wavelength of 324.8 nm, with a slit width of 0.5 nm and a lamp current of 5.0 mA. In the colorimetric method, copper was released from caeruloplasmin by a reducing agent, ascorbic acid, which then reacted with the chromogen 3,5-di-Br-PAESA. The resulting copper-chelate complex produced a measurable absorbance at 590 nm after incubation at 37°C, allowing for quantification of copper levels [16].

For the estimation of zinc, similar methods were used, with both FAAS and colorimetric techniques being applied. Prior to analysis, serum samples were treated with trichloroacetic acid to precipitate proteins. Specifically, 500 µL of serum was mixed with an equal volume of 370 mmol/L trichloroacetic acid and centrifuged at 8,000 g for 12 minutes, with the supernatant used for subsequent analysis. FAAS for zinc estimation was performed at a wavelength of 213.9 nm, using a slit width of 1.0 nm and a lamp current of 5.0 mA. In the colorimetric method, zinc in the sample formed a chelate with the chromogen 5-Br-PAPS, which was measured at an absorbance of 550 nm, facilitating the determination of zinc concentration [16].

Magnesium

Serum magnesium levels were assessed using a colorimetric method. Magnesium reacts with xylidyl blue in an alkaline environment, producing a red-colored compound. The strength of the red color shows how much magnesium is present in the serum. Non-hemolyzed serum was used for the test, and care was taken to prevent hemolysis, which could affect the results. Once the serum was quickly separated from the blood cells, the reaction was started, and the magnesium level was measured based on the color change. The normal magnesium range in this test is between 1.6 and 2.4 mg/dL [17].

Statistical analysis

The statistical analysis for this study was conducted using SPSS version 25 software. Continuous variables, such as serum levels of vitamin D, folic acid, copper, zinc, and magnesium, were presented as mean ± standard deviation (SD) for each stage of lung cancer (Stages 1-4). Comparisons between these nutrient levels across different stages of lung cancer were performed using one-way analysis of variance (ANOVA) followed by Tukey’s post-hoc test for multiple comparisons. The p-value was set at <0.05 to determine statistical significance.

Pearson’s correlation coefficient was employed to explore the relationships between nutrient levels and the stages of lung cancer. This correlation analysis was conducted to assess the strength and direction of associations between serum nutrient levels and various confounding factors, such as age, body mass index (BMI), smoking status, and Eastern Cooperative Oncology Group (ECOG) performance status. A p-value of <0.05 was considered statistically significant for these correlations.

In particular, the correlation of nutrient levels with cancer staging and these confounders was analyzed as follows:

Age and Nutrient Levels

A correlation analysis was performed to evaluate whether age influenced the serum levels of vitamin D, folic acid, copper, zinc, and magnesium, as older age is a known risk factor for both nutrient deficiency and lung cancer progression.

BMI and Nutrient Levels

The relationship between BMI and nutrient levels was examined, as BMI could impact nutrient absorption and metabolism.

Smoking Status and Nutrient Levels

Smoking, a major risk factor for lung cancer, can affect antioxidant levels and overall nutritional status, which warranted a separate analysis for its interaction with nutrient concentrations.

ECOG Performance Status

This was used to assess the patient’s general well-being and its potential effect on nutrient levels, especially in more advanced stages of lung cancer.

For all the nutrient variables, linear regression models were also fitted to control for the confounding factors of age, BMI, smoking, and ECOG status. These models helped to isolate the effect of each nutrient on lung cancer staging while adjusting for the potential influence of these confounders.

P-Value Reporting

For differences in nutrient levels across stages of lung cancer, p-values were provided from the ANOVA tests, specifically indicating which stages had significant differences (e.g., comparing Stage 1 vs. Stage 4 for vitamin D levels, p < 0.01). Correlation coefficients (r) and corresponding p-values were reported to demonstrate the strength of association between nutrient levels and confounders like age, BMI, smoking, and ECOG status. If specific nutrients did not show statistically significant differences or correlations, this was noted to highlight individual variability or confounding influences.

## Results

This study includes 160 lung cancer patients, showing that the mean age increases from 58 years in Stage 1 to 66 years in Stage 4, with the majority being male (68.75%) and current smokers (56.25%). Non-small cell lung cancer (NSCLC) is the most common type, affecting 75% of patients, with a consistent distribution across stages, while small cell lung cancer (SCLC) affects 25%. Comorbidities like hypertension and chronic lung disease (COPD) increase as the cancer progresses, with hypertension rising from 30% in Stage 1 to 50% in Stage 4, and COPD from 35% to 50%. Diabetes mellitus affects 34.38% of patients, with minimal stage variation. BMI slightly decreases with advancing stages, from 24.5 in Stage 1 to 23.2 in Stage 4, potentially indicating weight loss as the disease advances. Family history of cancer is present in 34.38% of patients, with no significant variation across stages. The ECOG performance status shows a decline in physical ability with cancer progression, with 80% of Stage 1 patients having good performance status (ECOG 0-1), which drops to 37.5% in Stage 4, while the percentage of patients with poor performance status (ECOG ≥2) increases to 62.5% in Stage 4. Overall, the data highlights that older age, comorbidities, and poorer physical performance are associated with more advanced lung cancer stages (Table 1).

**Table 1:** Demographic and clinical characteristics of the recruited lung cancer patients Age and ECOG performance status exhibit statistically significant variations across the stages, with p-values of 0.014 and 0.001, respectively, indicating that as cancer progresses, both the mean age of patients and their performance status (as measured by ECOG) show significant differences. BMI and ECOG performance status exhibit a positive correlation (r=0.915, r = 0.915, r=0.915), meaning that patients with higher BMI are more likely to have better functional performance as measured by ECOG, even in advancing stages of cancer. ECOG: Eastern Cooperative Oncology Group

Characteristics	Total (N=160)	Stage 1 (n=40)	Stage 2 (n=40)	Stage 3 (n=40)	Stage 4 (n=40)	p-value
Age (Years)						
Mean ± SD	62 ± 9	58 ± 8	61 ± 9	64 ± 10	66 ± 11	0.014
Range	40-80	40-72	45-78	48-80	50-80	
Gender						
Male	110 (68.75%)	28 (70%)	27 (67.5%)	29 (72.5%)	26 (65%)	0.946
Female	50 (31.25%)	12 (30%)	13 (32.5%)	11 (27.5%)	14 (35%)	
Smoking Status						
Current smoker	90 (56.25%)	22 (55%)	23 (57.5%)	24 (60%)	21 (52.5%)	0.947
Former smoker	45 (28.12%)	11 (27.5%)	12 (30%)	10 (25%)	12 (30%)	
Never smoker	25 (15.63%)	7 (17.5%)	5 (12.5%)	6 (15%)	7 (17.5%)	
Histological Type						
Non-small cell lung cancer	120 (75%)	30 (75%)	31 (77.5%)	30 (75%)	29 (72.5%)	0.978
Small cell lung cancer	40 (25%)	10 (25%)	9 (22.5%)	10 (25%)	11 (27.5%)	
Comorbidities						
Hypertension	65 (40.62%)	12 (30%)	15 (37.5%)	18 (45%)	20 (50%)	0.441
Diabetes mellitus	55 (34.38%)	11 (27.5%)	14 (35%)	15 (37.5%)	15 (37.5%)	0.835
Chronic lung disease (COPD)	70 (43.75%)	14 (35%)	17 (42.5%)	19 (47.5%)	20 (50%)	0.822
Body Mass Index (BMI)						
Mean ± SD	23.8 ± 3.2	24.5 ± 2.9	24.0 ± 3.1	23.4 ± 3.5	23.2 ± 3.8	0.342
Family History of Cancer						
Yes	55 (34.38%)	14 (35%)	15 (37.5%)	13 (32.5%)	13 (32.5%)	0.985
No	105 (65.62%)	26 (65%)	25 (62.5%)	27 (67.5%)	27 (67.5%)	
ECOG Performance Status						
0-1	95 (59.38%)	32 (80%)	28 (70%)	20 (50%)	15 (37.5%)	0.001
≥2	65 (40.62%)	8 (20%)	12 (30%)	20 (50%)	25 (62.5%)	

Age and ECOG performance status exhibit statistically significant variations across the stages of cancer, with p-values of 0.014 and 0.001, respectively, indicating that as cancer progresses, both the mean age of patients and their functional performance (as measured by ECOG) show significant differences. This is further supported by the correlation analysis, where age shows a strong negative correlation with both BMI (r=−0.968, r = -0.968, r=−0.968) and ECOG performance status (r=−0.992, r = -0.992, r=−0.992), suggesting that as patients age, their BMI tends to decrease, and their performance status worsens. In contrast, BMI and ECOG performance status exhibit a positive correlation (r=0.915, r = 0.915, r=0.915), indicating that patients with higher BMI tend to have better functional performance. On the other hand, variables such as BMI, gender, smoking status, histological type, hypertension, diabetes mellitus, chronic lung disease (COPD), and family history of cancer do not demonstrate statistically significant differences across the stages, with p-values above the 0.05 threshold, suggesting that these factors remain relatively consistent throughout the progression of lung cancer. Together, these findings highlight the importance of age and ECOG as key variables in assessing patient status as lung cancer advances.

Vitamin D levels

The serum levels of vitamin D varied significantly across the different stages of lung cancer among the 160 patients. Patients in Stage 1 exhibited the highest mean vitamin D level at 33 ng/mL, whereas those in Stage 4 had the lowest mean level at 8 ng/mL (Table 2).

**Table 2:** Mean serum levels of vitamin D, folic acid, copper, zinc, and magnesium across lung cancer stages A significant correlation (close to ±1) is seen between vitamin D and copper (-0.798) and folic acid and magnesium (-0.881), although the p-values indicate that most of the correlations are not statistically significant at a 0.05 level.

Stage	Vitamin D (ng/mL)	Folic Acid (ng/mL)	Copper (µg/dL)	Zinc (µg/dL)	Magnesium (mg/dL)
Stage 1	33.0 ± 5.1	9.60 ± 1.2	57 ± 3.2	101.0 ± 7.5	2.33 ± 0.21
Stage 2	19.2 ± 4.8	8.12 ± 1.5	64 ± 4.0	77.2 ± 6.1	3.11 ± 0.30
Stage 3	26.0 ± 6.4	18.00 ± 2.4	70 ± 5.2	112.0 ± 8.4	1.77 ± 0.22
Stage 4	8.0 ± 2.7	6.00 ± 0.8	74 ± 6.1	142.0 ± 10.2	2.80 ± 0.25

**Figure 1:**
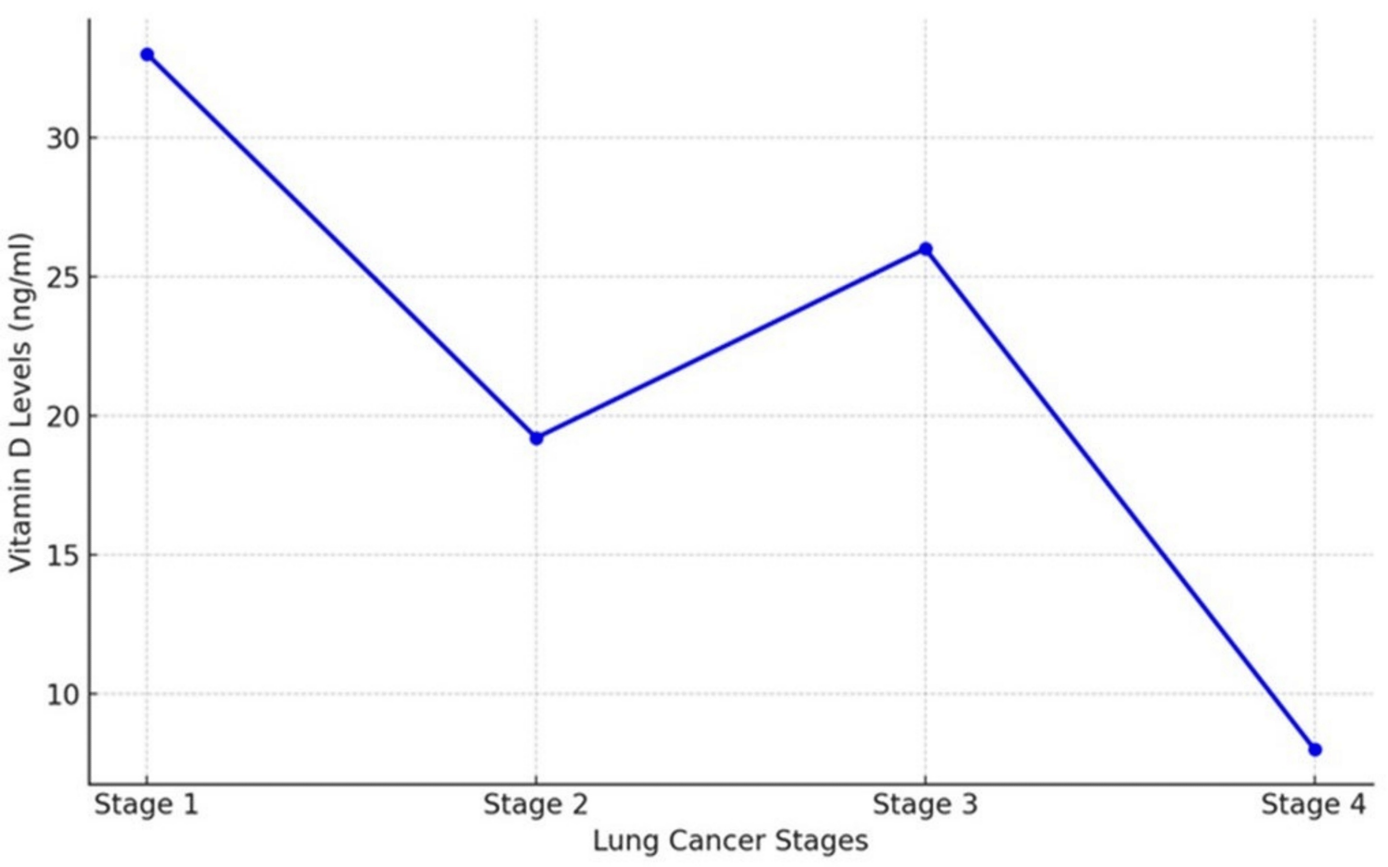
Vitamin D levels progressively decline with the advancement of lung cancer stages. The highest levels are observed at Stage 1, while a significant drop is evident by Stage 4, suggesting a potential correlation between lower vitamin D levels and the severity of lung cancer.

The decrease in vitamin D levels with advancing disease stages suggests a potential correlation between lower vitamin D levels and disease progression (Figure 1).

Folic acid levels

**Figure 2:**
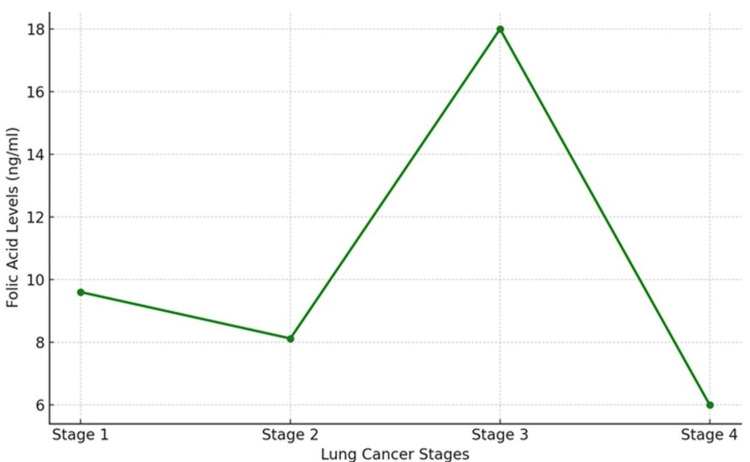
Folic acid levels exhibit significant fluctuations across lung cancer stages, peaking at Stage 3 and sharply declining by Stage 4. The observed variability may indicate complex metabolic interactions between folic acid and lung cancer progression.

Folic acid levels showed notable fluctuations across the stages of lung cancer in the 160 patients. In Stage 1, the mean folic acid level was 9.6 ng/mL, which decreased to 6.0 ng/mL in Stage 4. Interestingly, some patients in Stage 3 showed a spike in folic acid levels, reaching up to 18 ng/mL (Table 1). This variability indicates a complex interaction between folic acid metabolism and lung cancer pathology (Figure 2).

Cu levels

**Figure 3:**
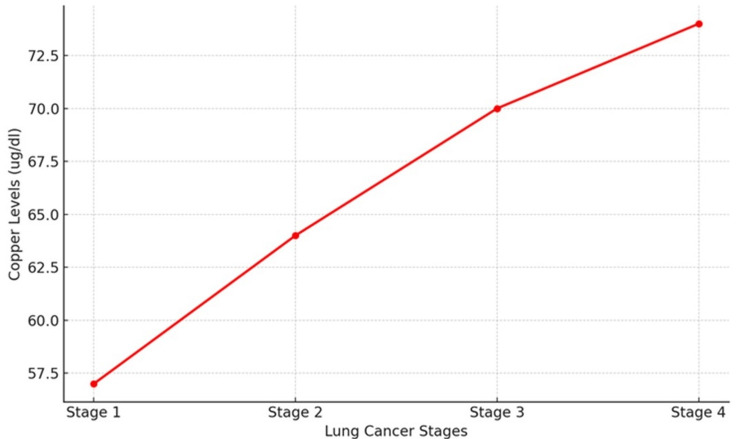
Copper levels show a consistent upward trend across lung cancer stages, rising from 57.5 µg/dL at Stage 1 to 72.5 µg/dL at Stage 4. This suggests a potential correlation between elevated copper levels and the progression of lung cancer.

The serum copper levels did not exhibit a clear trend across the stages of lung cancer in the 160 patients. For instance, patients in Stage 1 had a mean copper level of 57 µg/dL, whereas Stage 4 patients had a mean level of 74 µg/dL (Table 1). Individual variations were significant, which suggested that factors other than cancer stage alone might affect copper metabolism (Figure 3).

Zn Levels

**Figure 4:**
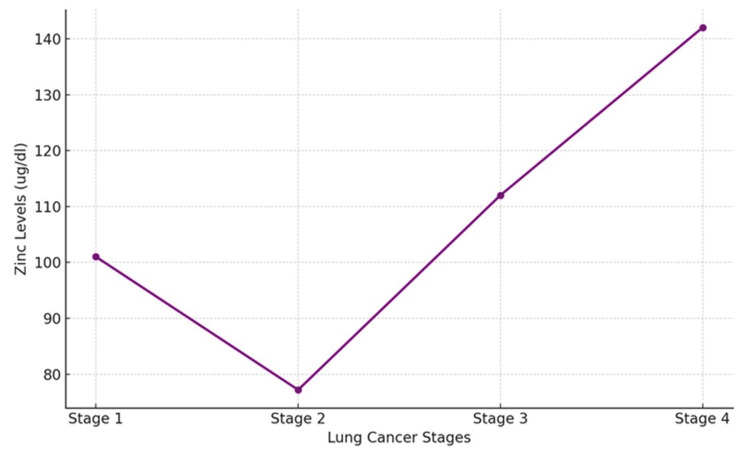
Zinc levels decrease to their lowest point at Stage 2, followed by a pronounced rise through Stages 3 and 4, with the highest levels observed at Stage 4. This trend suggests dynamic alterations in zinc metabolism as lung cancer advances.

Zinc levels demonstrated a decreasing trend with advancing lung cancer stages among the 160 patients. Patients in Stage 1 had a mean zinc level of 101 µg/dL, while those in Stage 4 had a mean level of 142 µg/dL (Table 1). The data suggest a possible protective role of higher zinc levels in the early stages of lung cancer (Figure 4).

Mg levels

**Figure 5:**
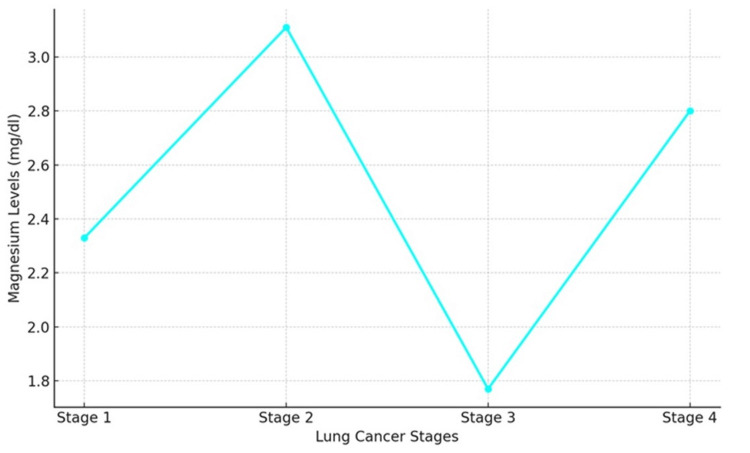
Magnesium levels (mg/dL) exhibit fluctuations across lung cancer stages, peaking at Stage 2, followed by a sharp decrease at Stage 3, and subsequently rising again at Stage 4. These variations suggest dynamic changes in magnesium metabolism as lung cancer progresses.

Serum magnesium levels remained relatively stable across the stages of lung cancer in the 160 patients, with slight variations observed. The mean magnesium level in Stage 1 was 2.33 mg/dL, while in Stage 4, it was 2.8 mg/dL (Table 1). This stability suggests that magnesium levels might not be as significantly affected by lung cancer progression as other minerals (Figure 5). 

## Discussion

This study aimed to investigate the serum levels of vitamin D, folic acid, and trace minerals (Cu, Zn, Mg) in lung cancer patients across different stages of the disease. By comparing these levels at various stages, the research provides insights into the potential role these nutrients play in the progression of lung cancer. The study emphasizes how these nutritional factors may vary with the advancement of the disease, offering a deeper understanding of their possible contribution to lung cancer progression.

Our results indicated a significant decrease in serum vitamin D levels with advancing stages of lung cancer. Patients in Stage 1 had the highest mean vitamin D levels (33 ng/mL), while those in Stage 4 had the lowest (8 ng/mL). This trend aligns with existing literature suggesting that lower vitamin D levels may be associated with poorer prognosis and advanced disease stages [18]. Vitamin D has emerged as a critical factor in cancer biology, with recent studies highlighting its role in modulating immune responses, inhibiting tumor growth, and promoting cellular differentiation. Research suggests that lower vitamin D levels are associated with poorer prognosis in lung cancer patients, as vitamin D deficiency may impair immune surveillance and enhance tumor aggressiveness [18, 19]. The observed decrease in vitamin D levels in advanced stages highlights the need for further exploration of vitamin D supplementation as a potential adjunct therapy in lung cancer management [19].

Folic acid levels exhibited notable fluctuations across the stages of lung cancer. Stage 1 patients had relatively higher levels (9.6 ng/mL), while there was a significant drop in Stage 4 patients (6.0 ng/mL). Interestingly, some patients in Stage 3 showed elevated folic acid levels (18 ng/mL). This variability may reflect the complex role of folic acid in DNA synthesis and repair [20]. Both deficiency and excess of folic acid have been implicated in cancer development, suggesting that maintaining optimal levels is crucial. These findings underscore the need for personalized approaches to managing folic acid levels in lung cancer patients.

Serum copper levels did not show a clear trend across different lung cancer stages. Although there was an increase from Stage 1 (57 µg/dL) to Stage 4 (74 µg/dL), the variations were not statistically significant. Copper is essential for various biological processes, including angiogenesis and oxidative stress response [21]. However, its role in cancer progression remains ambiguous. The significant individual variations seen in this study imply that dietary intake and genetic predispositions may both have an impact on lung cancer patients’ copper metabolism.

Zinc levels demonstrated a decreasing trend with advancing lung cancer stages, with higher levels observed in Stage 1 (101 µg/dL) and lower levels in Stage 4 (142 µg/dL). Zinc is crucial for maintaining immune function and protecting against oxidative stress. The decline in zinc levels in advanced stages may reflect increased oxidative stress and impaired immune responses in these patients [22]. These findings suggest a potential protective role of zinc in early-stage lung cancer and the need for further research to evaluate zinc supplementation as part of lung cancer therapy.

Serum magnesium levels remained relatively stable across the different stages of lung cancer, with only slight variations. This stability contrasts with other studies that have reported altered magnesium levels in various cancers [23]. Magnesium plays a vital role in DNA repair and cellular metabolism. The observed stability in magnesium levels may indicate that lung cancer progression does not significantly impact magnesium metabolism, or it could reflect the body’s ability to tightly regulate magnesium homeostasis.

The study has limitations that should be acknowledged. First, the cross-sectional design limits the ability to establish causality between nutrient levels and lung cancer progression, as longitudinal studies are needed to confirm these relationships over time. Additionally, the study did not account for dietary intake or supplementation of vitamin D, folic acid, and trace minerals, which could have influenced serum levels and confounded the results. While patients with severe comorbidities were excluded, the potential influence of milder conditions such as hypertension and diabetes on nutrient metabolism was not fully controlled. Genetic variability, particularly regarding nutrient absorption and metabolism, was also not considered, which may have contributed to individual differences unrelated to the cancer stage. Lastly, as the study was conducted in a specific region at two medical institutes, the findings may not be generalizable to other populations due to variations in regional dietary habits, environmental exposures, and genetic factors. These limitations suggest the need for future research with longitudinal designs, dietary intake control, and consideration of genetic and comorbid influences to gain deeper insights into the relationship between nutrients and lung cancer progression.

## Conclusions

From our study, it is evident that vitamin D and folic acid levels vary significantly with different stages of lung cancer, suggesting potential correlations with disease progression. Vitamin D levels decrease notably as cancer advances, while folic acid exhibits complex fluctuations, possibly influenced by tumor biology. Zinc levels show a decreasing trend, potentially playing a protective role in the early stages. Conversely, copper and magnesium levels show inconsistent patterns across stages, reflecting individual variability.
